# poFUT1 promotes uterine angiogenesis and vascular remodeling via enhancing the O-fucosylation on uPA

**DOI:** 10.1038/s41419-019-2005-3

**Published:** 2019-10-10

**Authors:** Dandan Zhang, Yu Yang, Caixia Liang, Jianwei Liu, Hao Wang, Shuai Liu, Qiu Yan

**Affiliations:** Department of Biochemistry and Molecular Biology, Dalian Medical University, Liaoning Provincial Core Lab of Glycobiology and Glycoengineering, 116044 Dalian, China

**Keywords:** Glycobiology, Infertility

## Abstract

Uterine angiogenesis and vascular remodeling play critical roles in determing the normal menstrual cycle and successful pregnancy. Poor uterine angiogenesis usually results in pregnancy failure. Protein O-fucosyltransferase 1 (poFUT1) is the key enzyme responsible for O-fucosylated glycan biosynthesis on glycoproteins. However, the dynamic expression and regulation of poFUT1 on the uterine angiogenesis and vascular remodeling remain unknown. Here, we showed that the enlargement of the vascular lumen in the secretory phase was greater than that in the proliferative phase of the uterine endometrium during menstrual cycle; whereas there was a narrower vessel lumen and fewer blood vessels in the decidua from miscarriage patients than in that from healthy pregnancy women. Additionally, the expression of poFUT1 was increased in the uterine endometrium during the secretory phase compared with that in the proliferation phase, and its expression was decreased in the uterus of miscarriage patients compared with that of the healthy pregnancy women. Using hESCs and a mouse model, we demonstrated that poFUT1 increased the O-fucosylation on uPA, and activated of the RhoA signaling pathway, thus facilitating uterine angiogenesis and vascular remodeling. We also provide evidence that poFUT1 promotes hESCs angiogenesis by the decreased stemness of hESCs. These findings reveal a new insight into the uterine angiogenesis and vascular remodeling. The study suggests that poFUT1 could be seen as a novel potential diagnostic and therapeutic target for miscarriage.

## Introduction

Uterine angiogenesis and vascular remodeling are critical events during menstrual cycle and pregnancy^1,2^. In the secretory phase of the menstrual cycle, the uterus is receptive to the implantation of the blastocyst by increasing vasculature and blood flow as well as uterine secretions^3^. In early pregnancy, the uterine growth is rapid and accompanied by the profound neovascularization and vascular remodeling in order to increase uterine blood flow, which delivers sufficient oxygen and nutrients to the uterus and to the embryo. When the feto-placenta vascular system becomes structurally and functionally competent, it can mediate the exchange of apparatus between the mother and fetus^4^. The steroid hormones (progesterone and estrogen) and cytokines play orchestral roles in regulating uterine angiogenesis. Inadequate vascular development and angiogenesis result in pregnancy pathologies or pregnancy failure, i.e., preeclampsia or fetal growth restriction^5–8^. Embryonic trophoblast cells are involved in placental vascular system formation. However, the features and regulation of uterine angiogenesis and vascular remodeling, especially at the post-translational level, remain largely unknown.

Protein glycosylation, one of the most common and important post-translational modification, is involved in many physiological and pathological processes, including reproduction and tumorigenesis^9,10^. Protein fucosylation is a type of glycosylation. According to the glycosylation sites of the polypeptides, fucosylation is incorporated as two major forms: N-fucosylation and O-fucosylation, which are catalyzed by fucosyltransferases (FUTs) and protein O-fucosyltransferases (poFUTs), respectively^11,12^. Two poFUTs (poFUT1 and poFUT2) have been identified till now. Studies have demonstrated that FUTs and fucosylation are biologically important during reproductive processes. Fucosylated Lewis X antigens promote cell–cell adhesion in early-stage embryos^13^. FUT9 promotes neural development, and the knockout of FUT9 results in the development of anxiety-like behaviors in mice^14,15^. The knockout of poFUT1 and poFUT2 are lethal to mice. We previously reported that poFUT1 facilitated trophoblast invasion^16^. However, little is known whether poFUT1 is associated with uterine angiogenesis and vascular remodeling.

Urokinase-type plasminogen activator (uPA), a serine protease, that harbors an EGF repeat. poFUT1 mainly transfers fucose from GDP-fucose to serine or threonine residues in epidermal growth factor-like (EGF) repeats formulate O-fucosylation^16–18^. uPA binds to urokinase plasminogen activator receptor (uPAR), a specific cell-surface receptor that restricts the uPA to the cellular environment, and then activated uPA converts zymogen plasminogen into the active serine protease plasmin, which in turn activates multiple matrix metalloproteinases (MMPs) and other growth factors^19,20^. The defucosylation of uPA can not bind to the cell surface accompanied by the abolishment of mitogenic activity^21^, suggesting that O-fucose is necessary for the ligand–receptor interactions and subsequent signal transduction events. uPA has been found to be in tumor growth, metastasis, and angiogenesis. Raghu et al. found that silencing uPA inhibited angiogenesis via the enhanced secretion of SVEGFR1 in the endothelial and glioblastoma cells^22^. The research also revealed that uPA promoted angiogenesis by attenuating PRH transcription factor activity and VEGFR expression^23^. uPA and uPAR expressions have been determined in the human first trimester decidua endometrium^24^. However, the role of uPA O-fucosylation regulated by poFUT1 during uterine angiogenesis and vascular remodeling remains poorly understood.

In the present investigation, we showed an enlargement of the vascular lumens in the secretory phase compared with that in the proliferative phase of the uterine epithelium during the menstrual cycle, whereas a narrower of vessel lumen and fewer blood vessels were observed in the decidual from miscarriage patients than in that from healthy pregnant women. Additionally, the expression of poFUT1 increased in the uterine epithelium of the secretory phase compared with that in the proliferation phase, whereas this expression was decreased in the uterus of miscarriage patient compared with that in healthy pregnancy women. Exploring hESCs and a mouse model, we demonstrated that poFUT1 increased O-fucosylation on uPA following the activation of the RhoA-signaling pathway, thus facilitating uterine angiogenesis and vascular remodeling.

## Materials and methods

### Tissue samples

The tissue samples of proliferative phase, secretory phase, early pregnancy, and miscarriage at the age of 25–38 were collected form The Second Hospital of Dalian Medical University (Dalian, China). The proliferative phase (*n* = 10), secretory phase (*n* = 10) and early pregnancy women were with regular menstrual cycle who were excluded from other gynecological abnormalities. The normal pregnant group (*n* = 10) and miscarriage group (*n* = 10) were from 7 to 10 pregnancy weeks, and confirmed by ultrasound detection. This study was approved by the Clinical Ethics Review Board of Dalian Medical University.

### Cell culture

Human endometrial stromal cells (hESCs) were obtained from Professor Haibin Wang (Xiamen University, China) as a gift. Human umbilical vein endothelial cells (HUVEC) were obtained from the American Type Culture Collection (ATCC, Manassas, VA, USA). hESCs and HUVECs were cultured in DMEM/F12 (Invitrogen, Grand Island, NY, USA) containing 10% FBS, 100 μg/ml penicillin, and 100 U/ml streptomycin (Sigma-Aldrich, St. Louis, MO, USA). The cells were cultured at 37 °C, 5% CO_2_ and 90% humidity, and the medium was changed every 48–72 h. To induce decidualization in vitro, hESCs were treated with DMEM/F12 with 2% FBS which containing 10 nM E_2_ (Selleck, Chemicals, USA), 1 μM medroxyprogesterone 17-acetate (MPA, Selleck), and 0.5 mM dibutyryl cAMP (dbcAMP, Selleck).

### Real-time RT-PCR

Total RNA was extracted by using Trizol reagent (Takara, Dalian, China) according to the protocol. A PrimeScript RT Reagent Kit (Takara) was used for reverse transcription. A total of 1 µg RNA was used for complementary DNA synthesis. Real-time polymerase chain reaction (PCR) was carried out with the SYBR Premix Ex Taq II Kit (Takara) on ABI Prism 7500 Detection system (Applied Biosystem, Foster City, CA). GAPDH was applied as a loading control. The primers of poFUT1, VE-cadherin, CD31, and GAPDH were as followings: poFUT1 Forward 5′-CAG CGA AGC CCA GAT AAG AAG-3′, Reverse 5′-CTG TAG GAA GCT CTG AAG GAA AT-3′; VE-cadherin Forward 5′-TGT GTC TTT ACA CCT CGC TGT TG-3′, Reverse 5′-TTC CCT GCC CCT CTC TGT T-3′; CD31 Forward 5′- CCG CAT ATC CAA GGT CAG CA-3′, Reverse 5′-CAC CTT GGT CCA GAT GTG TGA A-3′; GAPDH Forward 5′-TCC TGT TCG ACA GTC AGC CGC AT-3′, Reverse 5′-TGC AAA TGA GCC CCA GCC TTC TCC A-3′. Each experiment was performed at least three times.

### Western blot assay

Cells were lysed in RIPA lysis buffer containing protease inhibitor cocktail (Roche, Basel, Switzerland). The protein concentration was determined with a BCA kit (Pierce, Rockford, IL, USA). Equal protein was separated with 12% SDS–PAGE gel and electrotransferred onto nitrocellulose membranes (Millipore, Billerica, MA, USA). After blocking with 5% non-fat dry milk in TBST for 2 h. The membrane was incubated with poFUT1 (1:1000, Proteintech, Wuhan, China), VE-cadherin (1:500, Elabscience, China), CD31 (1:1000, Proteintech), p-LIMK (1:1000, Abcam), LIMK (1:1000, Abcam), p-cofilin (1:1000, Abcam), cofilin (1:1000, Abcam), Rho A-GTP (1:2000, Abcam), and Rho A (1:500, Abcam) antibody overnight at 4 °C. After washing with TBST, the membrane was incubated with horseradish peroxidase-conjugated secondary antibody (1:3000, Beyotime, Shanghai, China) for 1 h at room temperature. An enhanced chemiluminescence (ECL) detection system (Bio-Rad) was used to visualize immunoreactivity bands. The relative protein level was quantified by densitometry and normalized to GAPDH level using Image J software (NIH, Bethesda, MD, USA).

### Immunoprecipitation (IP)

IP was conducted with protein G agarose beads (Thermo Fisher Scientific, Waltham, MA, USA) according to the manufacturer’s instructions. Cells after transfection were lysed and incubated in IP lysis buffer (Beyotime) for 10 min at room temperature. The extracts were incubated with anti-uPA-antibody (2 μg/ml, Abcam, Cambridge, UK) at 4 °C overnight, and the immunoprecipitants were purified by protein G agarose beads with gentle rocking. The beads were washed for three times with extraction buffer and resuspended in 20 μl SDS-loading buffer. The whole cell lysates and immunoprecipitants were incubated at 70 °C for 10 min followed with western blot analysis.

### O-fucosylation assay

The Click-iT® Protein Reaction Buffer Kit (Invitrogen, Grand Island, NY, USA) was used to assess the level of O-fucosylation. In brief, 6-alkynyl fucose (6AF) (50 μM) was added in the cell culture medium for 48 h. Lysate solution 200 μl (1% SDS, 50 mM Tris–HCl, pH 8.0) was added to the cell culture and lysed on ice for 30 min to collect cells. Click reactions were performed as on both the labeled cell lysate to tag glycoproteins containing 6AF with biotinylated azide at room temperature for 1 h. The tagged cell lysate was analyzed by immunoblot.

### Immunofluorescence

Immunofluorescent staining was performed after fixing the cells with 4% paraformaldehyde for 20 min. After blocking with 3% BSA (Beyotime) for 1 h, the cells were incubated with poFUT1 (1:200), VE-cadherin (1:200) and CD31 antibody (1:200) overnight at 4 °C. After washing with PBS, the cells were incubated with TRITC-conjugated goat anti-mouse IgG (1:100, ZSGB-BIO, Beijing, China) and FITC-conjugated goat anti-rabbit IgG (1:100, ZSGB-BIO) for 1 h at room temperature. Then slides were incubated with DAPI (1:1000, Beyotime) for 10 min at room temperature. Images were obtained under a fluorescence microscope (Olympus BX83, Japan).

### Wound-healing assay

Cells were scratched with a pipette tip at 80–90% confluence and incubated for the indicated times. Images of cell migration were taken with an inverted microscope. The average extent of the wound closure for each group was quantified.

### Tube formation assay

The method is according to Nowak-Sliwinska et al. ^25^. Briefly, the matrigel (Becton Dickinson, Bedford, MA, USA) was pre-thawed at 4 °C overnight, and the pipette tips required for the experiment was pre-cooled. The cells subjected to the specific treatment were taken out, the serum-free medium was replaced, and then the cells were placed in an incubator for further 6 h. Using a pre-cooled tip, the matrix glue was placed in a 96-well plate to avoid air bubbles and incubate in a cell culture incubator for 1 h. Then, the treated cells were removed, the medium containing Cell Tracker Green (CMFDA, Life Technologies, Carlsbad, CA, USA) was replaced, and incubated in an incubator for 15–30 min. The cells were washed three times with PBS, then digested with trypsin and centrifuged for 5–8 min. The cells were resuspended in serum-free medium to a cell density of about 5 × 10^4^/ml. The cells were placed on the solidified Matrigel and placed in an incubator. After 6 h of incubation, the tube formation was observed under the microscope.

### Tissue immunofluorescence and immunohistochemistry

Paraffin-embedded human/mouse uterine tissues were prepared, followed by deparaffinization and rehydration. After antigen being exposed, tissue slides were incubated with 3% H_2_O_2_ for 10 min and goat serum for 30 min to block non-specific binding. Primary antibodies: poFUT1 (1:200) and CD31 (1:200) antibody were incubated overnight at 4 °C. Washing with PBS, TRITC-conjugated goat anti-rabbit IgG (1:100) and FITC-conjugated goat anti-mouse IgG (1:100) were incubated for 1 h at 37 °C following treatment with DAPI (1:1000) for 3 min. The immunofluorescent images were taken with an inverted microscope (Olympus, Tokyo, Japan). For immunohistochemistry assay, Primary antibodies CD34 (1:200, Abcam, USA) was applied at 4 °C overnight. After washing with PBS, slides were incubated with the second antibody for 45 min and visualized with diaminobenzidine. The slides were counterstained with hematoxylin, and the evaluation was performed. Images were captured under an inverted microscope.

### Animal experiments

Mice (Kunming White Crossing Line; 7–8 weeks) were purchased from the Experimental Animal Center of Dalian Medical University (Dalian, China). The procedures involved in the mouse studies were approved by the Institutional Review Board of Dalian Medical University. The mouse was maintained in controlled conditions (22–25 °C, 60% humidity, 14L:10D). Pregnant mouse was obtained by housing one female and one male mouse. The day found the vaginal plug was defined as gestation day 1. On day 1 of pregnancy at 09:00, mouse was anesthetized and 10 µl solution containing 1 nmol poFUT1 siRNA was injected into the right uterus horn while the left with scramble RNA in 10 μl normal saline. Samples of mouse endometrium from day 1 to day 8 were collected. The number of implanted embryos was counted and analyzed statistically.

### Statistical analysis

The analysis was performed using GraphPad Prism 6 statistical software, and each experiment was repeated three times. The results were expressed as mean ± SEM, and one-way analysis of variance was used between the comparison groups. There was a significant difference of *P < 0.05, **P < 0.01 was a significant difference, and ***P < 0.001 was extremely significant.

## Results

### Expression of poFUT1 and features of angiogenesis and vascular remodeling in the uterine endometrium of different functional states

**Fig. 1:**
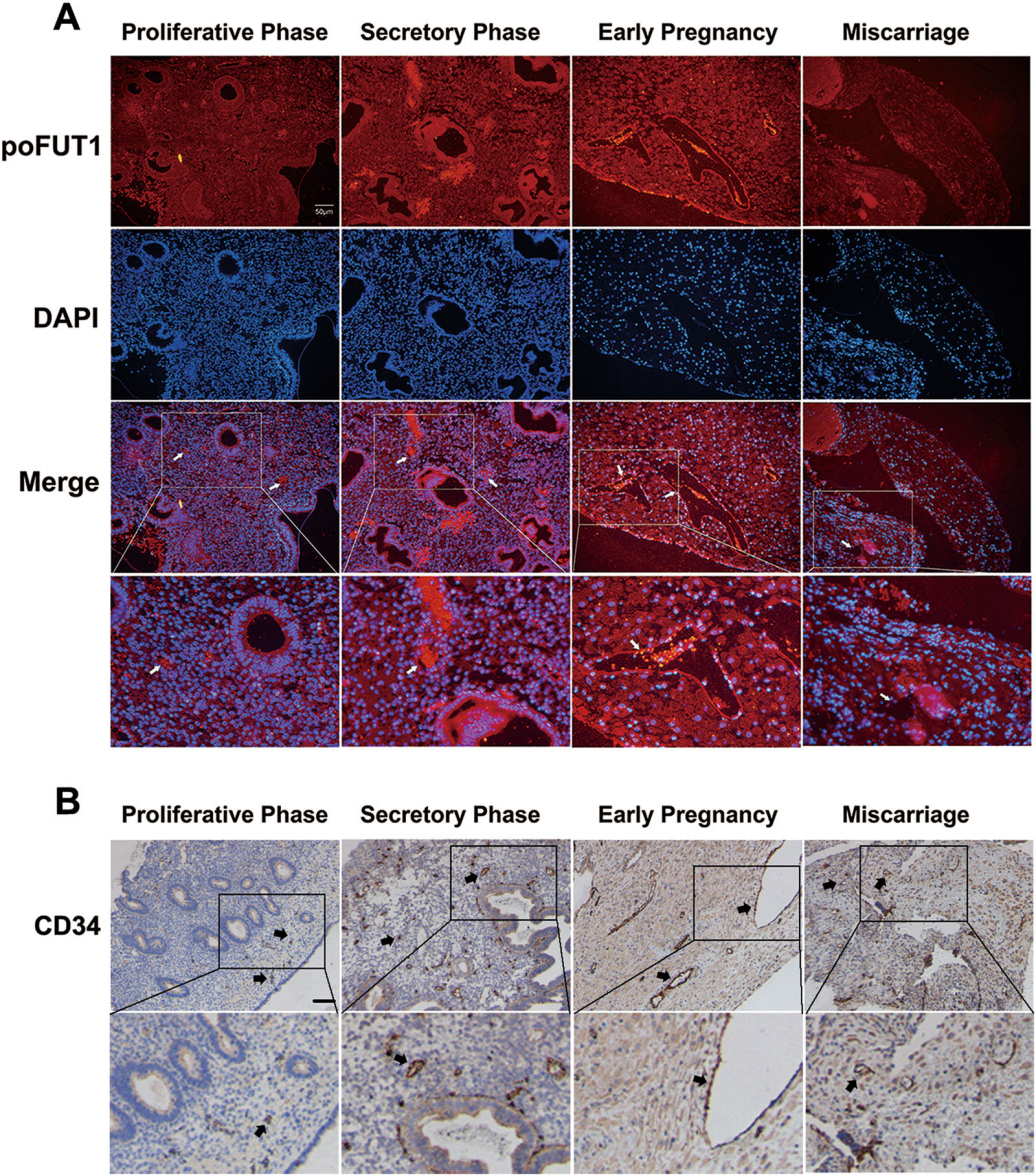
Expression of poFUT1 and features of angiogenesis and vascular remodeling in the uterine endometrium of different functional states. **a** Immunofluorescent staining of poFUT1 expression in the human uterine from proliferative phase, secretory phase, early pregnant women and miscarriage patients. White arrow: vascular. **b** Immunohistochemistry staining of CD34 expression in the human uterine vascular. Black arrow: vascular

poFUT1 was expressed in the uterine endometrium, and the immunofluorescent staining showed the higher levels of poFUT1 in the secretory phase compared with those in the proliferative phase, with the highest expression in the decidual phase of early pregnancy; whereas the levels of poFUT1 were decreased in miscarriage patients (Fig. 1a). We also found that poFUT1 was expressed on the vascular endothelial cells (Fig. 1a). Uterine vascular maturation is known to occur during the secretory phase of the menstrual cycle; thus, we next compared the structural features in the vessels and the molecular expression of angiogenesis in the secretory and proliferative phases. CD34 as the vascular marker was detected. As shown in Fig. 1, enlargement of the vascular lumen was observed in the secretory phase compared with that in the proliferative phase using immunohistochemical staining. Furthermore, in early pregnancy, increased vascular formulation was observed in the decidual phase, whereas narrower vascularization was observed in miscarriage patients compared with women in the early pregnancy phase (Fig. 1b).

### poFUT1 plays a major role in human hESCs angiogenesis and vascular remodeling during early pregnancy

**Fig. 2:**
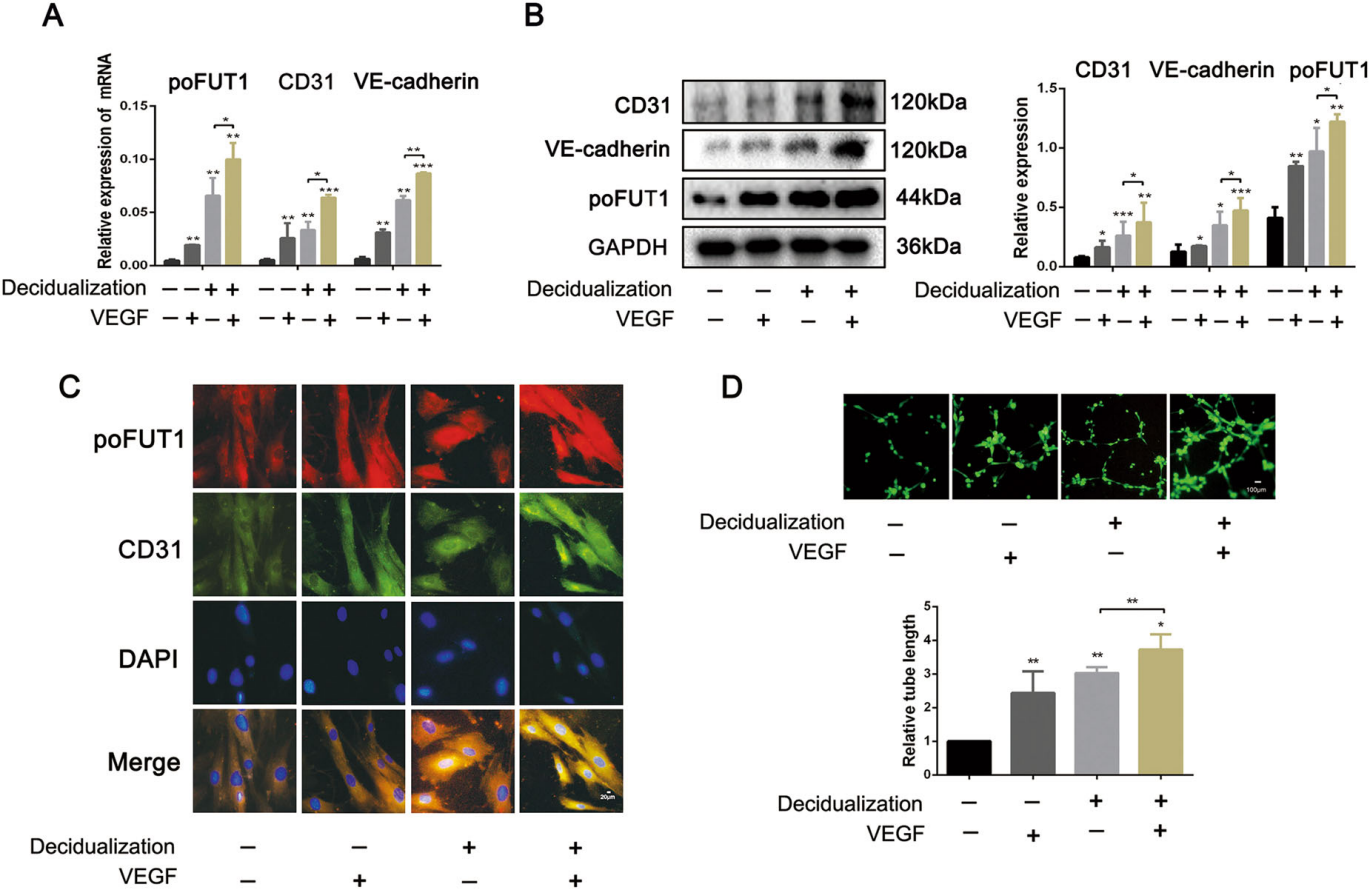
Increased levels of poFUT1 and increased angiogenesis potential in artificial decidua hESCs. hESCs were treated with artificial decidua element (10 nM E_2_, 1 μM MPA, 0.5 mM cAMP) or VEGF for 72 h. **a**, **b** Real-time PCR (**a**) and western blot (**b**) analyses of poFUT1, CD31, and VE-cadherin expression. **c** Immunofluorescent staining of poFUT1, CD31, and VE-cadherin in hESCs. **d** Tube formation assay of hESC angiogenesis. Significance was indicated by *P < 0.05, **P < 0.01, ***P < 0.001

Based on the observations of angiogenesis changes and poFUT1 expression in the uterine tissues from menstrual cycle, early pregnancy and miscarriage, we further explored the role of poFUT1 in the angiogenesis and vascular remodeling using hESCs. First, we analyzed the expression of poFUT1 and vascular endothelial cell special marker (CD31) in hESCs after artificially induced decidualization (hormone decidual stimulus (17β-estradiol (E2), medroxyprogesterone acetate (MPA), and cAMP (EPC)) or VEGF. Quantitative real-time PCR (Fig. 2a) and western blot (Fig. 2b) results revealed that, compared to the untreated hESCs, poFUT1 and CD31 and VE-cadherin mRNA and protein were higher in decidualized hESCs. Similarly, poFUT1 and CD31 and VE-cadherin mRNA and protein were also increased in VEGF treatment group. Immunofluorescent staining showed a stronger density for staining of poFUT1 and CD31 in artificially induced decidualized hESCs than in undecidualized hESCs. Moreover, we observed the role of decidualization on hESCs tube formation ability, and the results suggested that artificial decidualization or VEGF-treated hESCs showed greater possibility of tube formation (Fig. 2d).

**Fig. 3:**
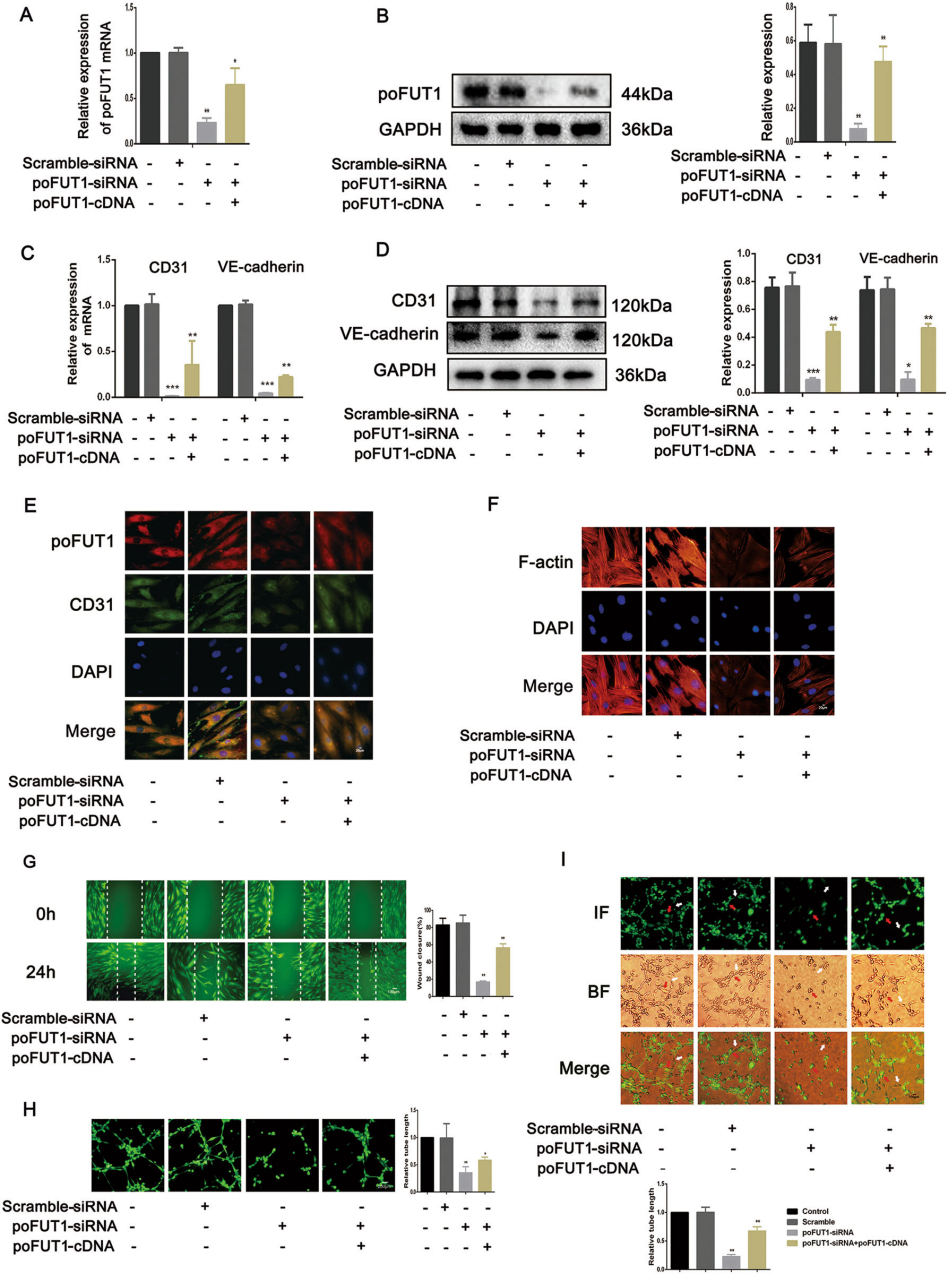
poFUT1 is involved in hESCs motility and angiogenesis and vascular remodeling ability. hESCs transfected with scramble-siRNA, poFUT-siRNA, or co-transfected with poFUT1-siRNA and poFUT1-cDNA. **a**–**d** Real-time PCR (**a**, **c**) and western blot (**b, d**) analyses of poFUT1, CD31, and VE-cadherin at the relative transcript level and protein level. **e** Immunofluorescent staining of poFUT1, CD31, and VE-cadherin in hESCs. **f** F-actin cytoskeleton change of hESCs. **g** Wound-healing assay detected hESC motility. **h** Tube-formation assay analyzes hESC angiogenesis ability. **i** Tube-formation assay analyzes coangiogenesis of hESCs and HUVECs. Significance was indicated by *P < 0.05, **P < 0.01, ***P < 0.001

To explore the regulatory role of poFUT1 in the uterine angiogenesis, we transfected poFUT1 siRNA or co-transfected poFUT1 siRNA and cDNA into hESCs. As shown in Fig. 3a, b, poFUT1 siRNA inhibited poFUT1 expression at mRNA and protein levels; whereas poFUT1 cDNA partially restored the poFUT1 expression. Correspondingly, silencing poFUT1 inhibited the expression of vascular endothelial cell markers, including CD31 and VE-cadherin (Fig. 3c–e). Angiogenesis and vascular remodeling are one of the main patterns in decidual angiogenesis during early pregnancy. Cell migration and invasion ability usually serve as detection markers for angiogenesis. In healing and transwell assays, poFUT1 siRNA markedly decreased hESCs migration and invasion potential. However, poFUT cDNA alleviated the inhibitiory effect (Fig. 3g, h). We also detected the effect of poFUT1 on the expression of the skeleton F-actin in hESCs. Immunofluorescence staining revealed that poFUT1 siRNA hampered F-actin bundle formation and cytoskeletal reorganization in hESCs; whereas poFUT1 cDNA restored the hampered effect (Fig. 3f). We found that up-expression of poFUT1 by artificial decidualization promoted hESCs angiogenesis. Most importantly, poFUT1 is also involved in vascular remodeling in hESC and HUVEC co-culture models in vitro. As shown in Fig. 3i, hESCs could migrate among the HUVECs, and form tubes with HUVECs. poFUT1 siRNA inhibited the tube formation of hESCs and HUVECs; whereas poFUT1 cDNA reversed the inhibition. Taken together, these findings suggested that poFUT1 is necessary for hESCs in both angiogenesis and vascular remodeling; its deficiency significantly hampers angiogenesis and vascular remodeling.

### Increased O-fucosylation on uPA by poFUT1 activates the Rho signaling pathway, and further promotes hESCs angiogenesis

**Fig. 4:**
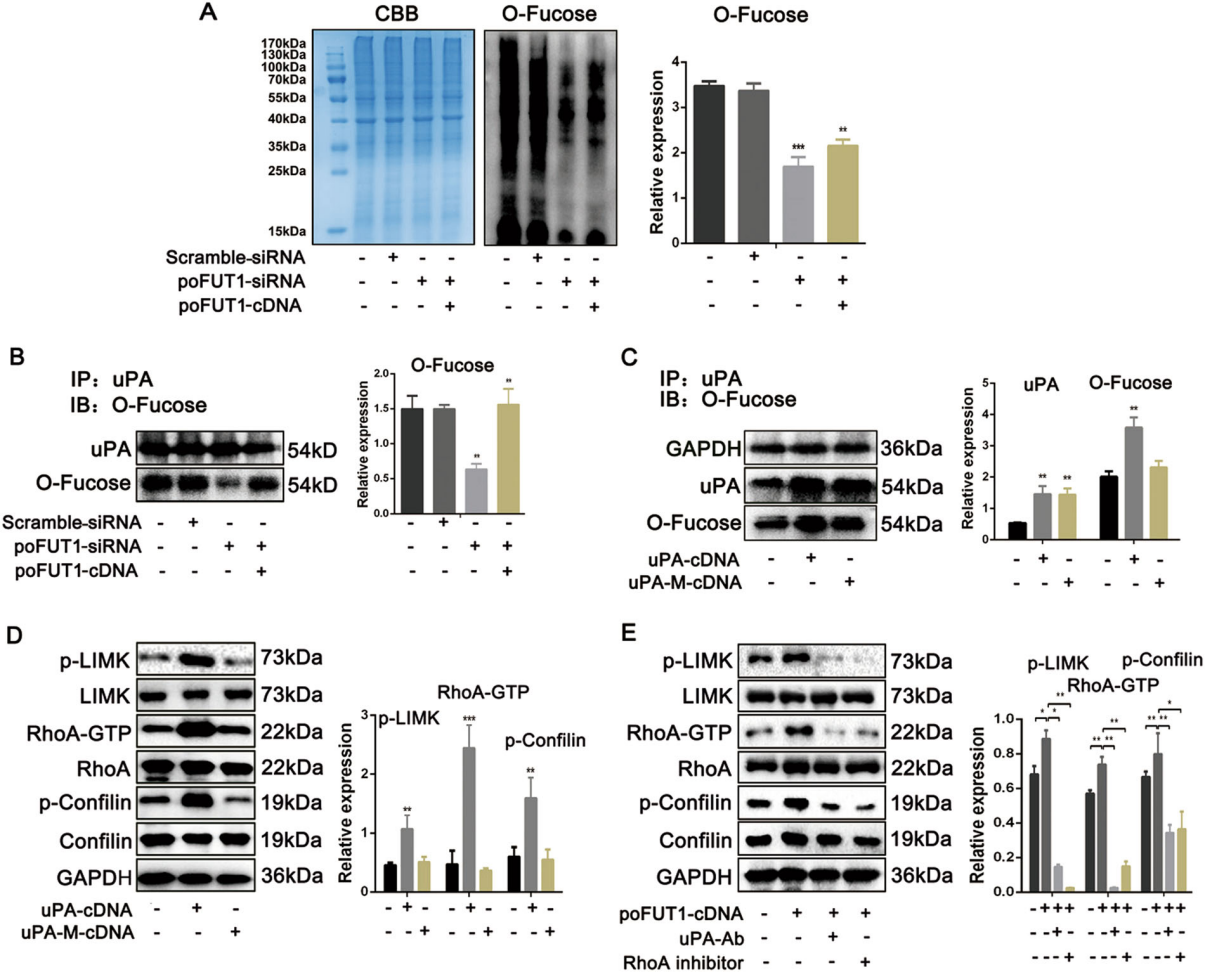
poFUT1 increase the biosynthesis of O-fucose on uPA and activated Rho signaling pathway. **a** O-fucose detection assay analysis of O-fucosylation on hESCs after transfection with scramble-siRNA, poFUT-siRNA, or co-transfection with poFUT1-siRNA and poFUT1-cDNA. **b** Immunoprecipitation and western blot analysis of O-fucosylation on uPA in hESCs after transfected with scramble-siRNA, poFUT-siRNA, or co-transfected with poFUT1-siRNA and poFUT1-cDNA. Immunoprecipitation (IP): anti-uPA antibody pulled down protein. Immunoblot (IB): detection of O-fucosylation by O-fucos detection assay. **c** IP and western blot analysis of O-fucosylation on uPA in hESCs transfected with uPA-cDNA or uPA-M-cDNA. uPA-M-cDNA: uPA mutation cDNA. **e** Western blot and statistical analysis of p-LIMK, LIMK, Rho-GTP, RhoA, p-Confilin, Confilin in hESCs transfected with uPA-cDNA or uPA-M-cDNA. **f** Western blot and statistical analysis activation of RhoA signaling pathway in hESCs transfected with poFUT1-cDNA or with uPA antibody, RhoA inhibitor blocking. Significance was indicated by *P < 0.05, **P < 0.01, ***P < 0.001

poFUT1 is mainly responsible for adding the fucose residues to specific glycoproteins through O-linkage. The O-linked glycoproteins play critical biological functions. To reveal the underlying mechanism of poFUT1 in angiogenesis, we first analyzed the O-fucosylation. As illustrated in Fig. 4a, poFUT1 siRNA inhibited O-fucose addition; whereas poFUT1 cDNA alleviated the inhibitory effect. uPA is an O-fucosylation glycoprotein, that is usually involved in embryo implantation and tumor metastasis. Herein, we further detected alterations in uPA O-fucosylation. The results showed that downregulation of poFUT1 by poFUT siRNA inhibited O-fucosylation on uPA, and the upregulation of poFUT1 by poFUT1 cDNA enhanced O-fucosylation on uPA (Fig. 4b). The Thr34 is the O-fucosylation site on uPA. Therefore, we constructed a uPA Thr34 mutation plasmid (uPA-M-cDNA) to ascertain the O-fucosylation on uPA in hESCs. As expected, uPA cDNA elevated O-fucosylation on uPA in hESCs. Oppositely, uPA mutation cDNA transfection only increased uPA protein expression, but not O-fucosylation on uPA (Fig. 4c). These results indicates that the regulatory role of poFUT1 on the biosynthesis of O-fucosylation on uPA in hESCs.

To clarify the signaling cascade underlying poFUT1 and O-fucosylation on uPA, the activation of the Rho-signaling pathway was detected. We compared the RhoA-signaling pathway activation after uPA cDNA or uPA mutation cDNA transfection. The results clearly showed that the levels of p-LIMK, RhoA-GTP, and p-Cofilin were induced in the uPA cDNA transfection group, whereas uPA mutation cDNA transfection displayed no obvious differences (Fig. 4d). We also noted that poFUT1 cDNA increased p-LIMK, RhoA-GTP, and p-Cofilin expression, and uPA-Ab or RhoA inhibitor treatment weakened the activation of RhoA-signaling pathway (Fig. 4e).

**Fig. 5:**
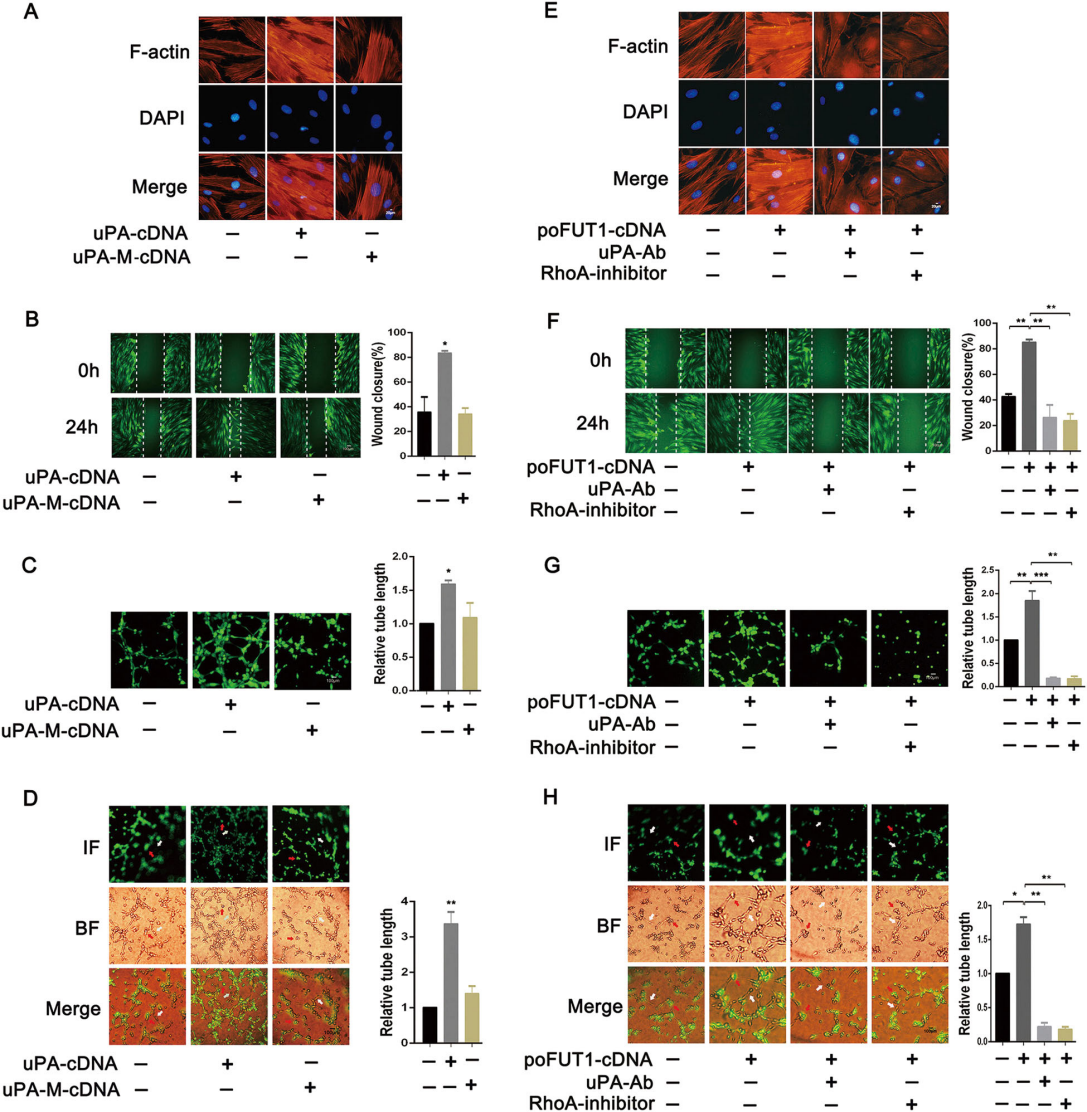
poFUT1 increase hESC angiogenesis and vascular remodeling by O-fucosylation on uPA and RhoA signaling pathways. hESCs transfected with uPA-cDNA or uPA-M-cDNA. hESCs transfected with poFUT1-cDNA or with uPA antibody, RhoA inhibitor blocking. **a, e** F-actin cytoskeleton change of hESCs. **b, f** Wound healing assay detected hESCs motility. **c**, **g** Tube formation assay analyzes hESCs angiogenesis ability. **d**, **h** Tube formation assay analyzes coangiogenesis of hESCs and HUVEC. Significance was indicated by *P < 0.05, **P < 0.01, ***P < 0.001

To verify the roles of poFUT1-uPA-Rho in hESCs angiogenesis and vascular remodeling, we detected the changes in cell mobility and angiogenesis using cell migration, invasion, and tube formation assays. As expected, uPA cDNA transfection promoted, whereas uPA mutation cDNA transfection showed no obvious alterations in F-actin bundle formation (Fig. 5a). poFUT1 cDNA increased, and uPA-Ab or RhoA-inhibitor weakened the cytoskeletal reorganization (Fig. 5e). Similarly, uPA cDNA increased hESC migration and invasion ability. However, uPA mutation cDNA transfection had no effect on hESC motility (Fig. 5b, f). The tube formation assay demonstrated that activation of RhoA-signaling pathway by uPA cDNA or poFUT1 cDNA not only promoted tube formation in hESCs (Fig. 5c, g), but also increased the co-tube formation of hESCs and HUVECs ability (Fig. 5d, h). These findings revealed that poFUT1 increased O-fucosylation on uPA, activated the RhoA-signaling pathway, and further promoted endometrial angiogenesis and vascular remodeling.

### poFUT1 facilitates the stemness characteristics of hESCs

**Fig. 6:**
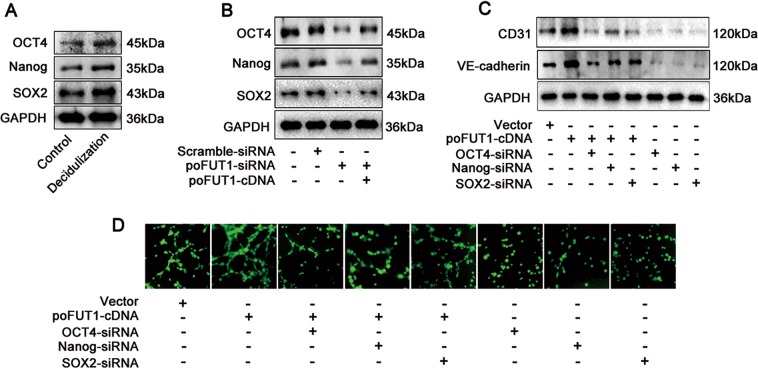
poFUT1 increase the stemness characteristics of hESCs. **a** Western blot analysis of OCT4, Nanog, and SOX2 expression in hESCs treated with artificial decidua element (E_2_, MPA, cAMP). **b** Western blot analysis of OCT4, Nanog, and SOX2 expression in hESCs transfected with scramble-siRNA, poFUT1-siRNA or cotransfected with poFUT1-siRNA and poFUT1-cDNA, respectively. **c** Western blot analysis of CD31 and VE-cadherin in hESCs. **d** Tube formation assay analysis of angiogenesis in hESCs

Based on the finding that poFUT1 promoted hESC angiogenesis, we subsequently asked why hESCs could transform into vascular-like cells, and participate in angiogenesis. Decidualization of the human uterine endometrium involves the dramatic morphological and functional differentiation of hESCs. The fibroblast-like hESCs transform into epithelioid cells, with the characteristics of secretory cells. Thus, we hypothesized that stem cell characteristics that facilitate hESCs transformation into vascular endothelial cells. To confirm this hypothesis, we detected the expression of stem cell makers (OCT4, Nanog, and SOX2) in hESCs, examined the relationship between poFUT1 and stem cell markers, and further observed stem cell characteristics and angiogenesis. As shown in Fig. 6a, decidualized hESCs expressed less stem cell markers compared with the undecidualized hESCs. poFUT1 siRNA downregulated, whereas poFUT1 cDNA upregulated the expression of OCT4, Nanog, and SOX2 (Fig. 6b). We next determined the role of OCT4, Nanog, and SOX2 on hESC angiogenesis. Compared with the control group, poFUT1 cDNA increased the expression of CD31 and VE-cadherin. In contrast, OCT4, Nanog, and SOX2 siRNA decreased CD31 and VE-cadherin expression (Fig. 6c). Consistently, increased OCT4, Nanog, and SOX2 by poFUT1 cDNA promoted hESC tube formation. Contrarily, OCT4, Nanog, and SOX2 siRNA inhibited hESC tube formation (Fig. 6d). These findings suggest poFUT1 is involved in hESCs angiogenesis by alternating the stem cell character of hESCs, but the exact mechanism of poFUT1 and stem cell characteristics needs further study.

### poFUT1 promotes decidual angiogenesis in a mouse model

**Fig. 7:**
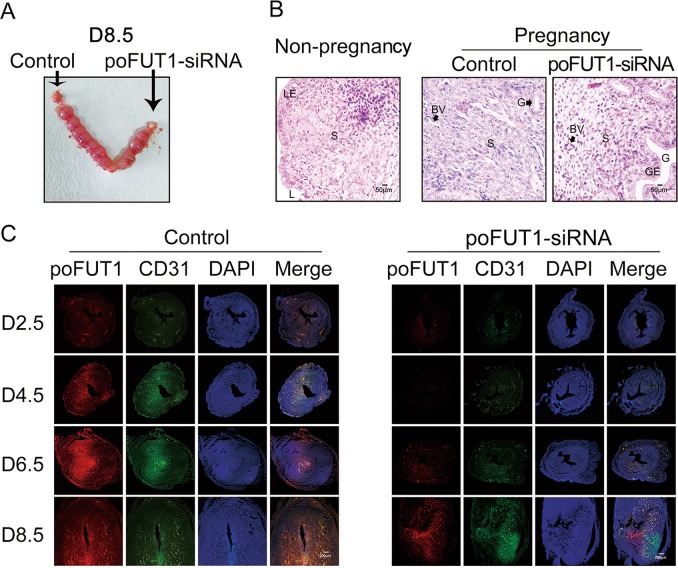
poFUT1 promote decidual angiogenesis in mouse model. Mouse uterus was injected with control (Scramble-siRNA) or poFUT1-siRNA on day 1 of pregnancy. **a** Number of implanted embryos in the uterus on day 8.5 pregnancy. **b** HE staining in the uterine endometrium of non-pregnant, Day 4 of pregnancy and Day 4 of pregnancy combined with poFUT-siRNA injection. **c** Immunofluorescent analyses of poFUT1, CD31 expression in the decidua on Day 2.5, Day 4.5, Day 6.5, and Day 8.5

After testing that poFUT1 downregulation hampered endometrial vasculature in vitro, we further investigated whether poFUT1 siRNA inhibited uterine angiogenesis and embryo implantation by applying a mouse model. poFUT1 siRNA or scramble siRNA was administered into the mouse uterus on D1 of pregnancy. The uterine samples were collected for detection on D2.5, D4.5, D6.5, and D8.5, and the embryo implantation rate was calculated on D8.5. The embryo implantation rate demonstrated that poFUT1 siRNA administration significantly impaired embryo implantation (Fig. 7a). Compared with the non-pregnant mouse uterine and endometrium, the pregnant mouse decidua showed more quantity and an enlarged vessel lumen, whereas the poFUT1 siRNA administration group showed fewer vessels and a smaller lumen than did the early pregnancy group (Fig. 7b). Furthermore, the staining of poFUT1 and CD31 were weaker in the poFUT1 siRNA administration group than that in the control group on D4.5, D6.5, and D8.5 (Fig. 7c), suggesting that poFUT1 siRNA decreased decidual angiogenesis and hampered decidual function, which further cause embryo implantation failure. Taken together, these findings implied that poFUT1 and O-fucosylation are necessary for uterine angiogenesis; their deficiency significantly hampers decidual angiogenesis.

## Discussion

Enoangiogenesis in uterine decidua during early pregnancy is essential for the female reproductive system and successful embryo implantation^26^. The new vascular system develops and matures by angiogenesis and vascular remodeling in the human endometrium to supply oxygen and nutrition to both the mother and the fetus^4^. Human endometrial angiogenesis is regulated by multitude factors, including hormones (P4, E), cytokines, and growth factors (VEGF, sVEGFR-1), etc.^27–29^. Lower maternal level of VEGF was observed in unexplained recurrent miscarriage compared to those in healthy and pregnant women^30^. The secretory phase of the uterus is the vascular maturation period, which expresses more VEGF than in the proliferative phase during the menstrual cycle. sFlt-1 and sEng damage endothelial cell vascular functions and induce complications in preeclampsia patients^31^. A previous study suggested that angiogenesis is involved in uterine function. In this study, we detected the features of angiogenesis and vascular remodeling in the uterine endometrium from different stages of the menstrual cycle, in early pregnancy women and miscarriage patients. Immunochemical staining revealed that the lumens were enlarged in the secretory phase compared with that in the proliferation phase during the menstrual cycle, whereas the narrower of vessel lumens and fewer of blood vessels in the decidua from miscarriage patients than in healthy pregnant women were observed (Fig. 1). In a mouse model, the number of vessels was increased with the pregnancy progression, whereas the decreased number of vessels in the decidual endometrium was associated with less embryo implantation (Figs. 1 and 7). These findings imply that impaired uterine angiogenesis hampers decidual differentiation, and even leads to embryo implantation failure.

FUTs are essential for reproduction in a spatiotemporal specificity manner. The accumulated evidence shows that FUTs participated in sperm–oocyte recognition, uterine receptivity formation, and trophoblast invasion at the fetal–maternal interface^32,33^. Lewis X, catalyzed by FUT, is a ligand for a major class of ZP3-binding sites on mouse sperm^33^. FUT4 contributed to the establishment of a receptive uterine endometrium receptivity^34^. Our previous results also demonstrated that poFUT1 expression was decreased in placental villi from miscarriage patients, and silencing poFUT1 suppressed the proliferation and invasion of JAR cells through inactivating MAPK and PI3K/Akt-signaling pathways^16,35^. These findings suggest that poFUT1 may be responsible for embryo implantation failure. Here, we further explored the expression and role of poFUT1 in uterine endometrium angiogenesis. We found that the expression of poFUT1 was higher in the uterine endometrium of secretory phase than that of the proliferative phase in the uterine endometrium, and poFUT1 levels were also higher in the decidual of early pregnant women decidua than in that of miscarriage patients (Fig. 1a). An elevated level of poFUT1 was also observed in pregnant decidua compared to the unpregnant uterus in a mouse model (Fig. 7). The dynamic changes in poFUT1 are similar to angiogenesis. Thus, poFUT1 is possible for uterine endometrium angiogenesis. Using hESCs, we found that downregulation of poFUT1 by specific siRNA transfection inhibited cell migration, invasion, and tube formation; in contrast, poFUT1 cDNA rescued this inhibitory effect. In summary, poFUT1 promotes hESCs angiogenesis and vascular remodeling.

Fucosylation usually affects the glycoprotein biological functions; including cell adhesion, migration and signaling transduction^36^. The elevated core fucosylation by FUT8 promoted non-small cell lung cancer cell migration and invasion^37,38^. The deletion of α-1,3-fucosylation by FUT4 siRNA inhibited A431 cell migration. In this study, we first identified that O-fucosylation biosynthesis could be regulated by poFUT1 in hESCs (Fig. 4). Furthermore, poFUT1 siRNA inhibited O-fucosylation on uPA. uPA is a kind of O-fucosylation glycoprotein, and Thr38 is the O-fucosylation site. The domain of uPA from residues 34 to 57 is responsible for binding uPAR. We found that a Thr38 mutation in uPA abolished the binding effect of uPA to uPAR (Fig. 4). Therefore, poFUT1 alternates uPA function by regulating O-fucosylation on uPA. uPA is usually responsible for cell mobility and, is associated with tumor metastasis and angiogenesis. The Rho-signaling pathway mainly mediates F-actin resemble and cell motility. Daniela Alfano et al. indicated that RhoB was a key regulator of uPA/uPAR signaling in cell adhesion, migration, and invasion^39^. Here, we revealed that the increased uPA activated the RhoA-signaling pathway, further promoting hESCs mobility, cytoskeletal reorganization, and tube formation, especially the co-tube formation of hESCs and HUVECs. Collectively, our findings reveal that poFUT1 increases O-fucosylation on uPA, activates RhoA-signaling pathway, and facilitated hESCs angiogenesis and vascular remodeling.

**Fig. 8:**
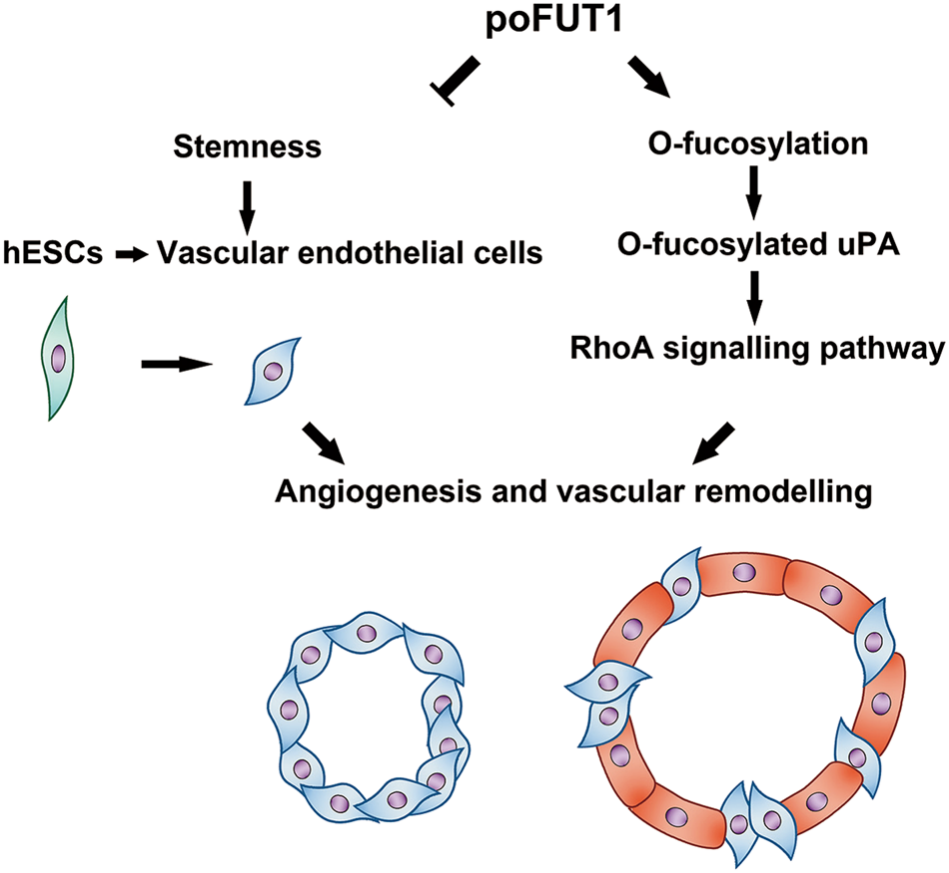
Summary of poFUT1 in uterine angiogenesis and vascular remodeling during pregnancy

Angiogenesis is common and vital process in the growth and development of the individuals. During the embryonic developmental period, angiogenesis is the embryonic formation of endothelial cells from mesoderm cell precursors. After birth, angiogenesis is the physiological process through which new blood vessels are formed from the pre-existing vessels^40^. In utero, vascular endothelial cell proliferation is associated with sprouting angiogenesis^41^. During placentation, some extravillous trophoblasts (EVT) cells enter uterine arteries as endovascular trophoblasts that participate in new maternal blood vessel formation^42,43^. Because uterine decidualization involves the morphological and functional differentiation of hESCs, we aimed to determine whether the hESCs showed stem cell properties that participate in angiogenesis. The results demonstrated the hESCs expressed stemness markers (OCT4, Nanog, SOX2), and silencing poFUT1 by poFUT1-siRNA decreased the expression of OCT4, Nanog, and SOX2, hampering tube formation in hESCs (Fig. 6). These findings imply that poFUT1 controls angiogenesis by changing hESCs stemness characteristics, and the concrete mechanism requires further study.

In summary, we revealed that fewer vessels and narrower vascular lumens were observed in the uterine endometrium of miscarriage patients than in early pregnant women. Additionally, poFUT1 was decreased in the uterine endometrium of miscarriage patients compared with that in early pregnant women. Moreover, we provided novel evidence showing that poFUT1 increased O-fucosylation on uPA, which further activated RhoA-signaling pathway, promoted uterine angiogenesis, and vascular remodeling, thus facilitating embryo implantation (Fig. 8). A better understanding of uterine endometrium angiogenesis and vascular remodeling may help develop diagnostic biomarkers and the related targeted therapies for the clinical treatment of miscarriage patients.
